# Selections

**Published:** 1891-08

**Authors:** 


### Selections.

### ETHER OR CHLOROFORM?

Dr. Julliard, after narrating a case in which death occurred during etherization, gives 20 cases of death by chloroform, 17 of those being published for the first time. Of the 20, no fewer than 15 occurred before the operation had commenced. He then sums up his argument by stating: (1) that any one who is anæsthetized by choloroform or by ether risks his life; and (2) that the risk is five times less with ether than with chloroform. If, then ether is less dangerous, why is it not preferred? This leads Dr. Julliard to discuss the objections to ether, and his fairness is indicated by his careful consideration of no fewer than eighteen objections: (1) Ether is disagreeable. This is a matter of taste, and is hardly worthy of consideration, in view of the risk. (2) Ether is difficult to administer, requiring complicated apparatus. Dr. Julliard gives it with a very simple mask containing gauze and flannel, and covered with macintosh cloth. (3) Ether is less active than chloroform, and the anæsthetic effect is more slowly produced. True; but the little delay is more than compensated by the additional safety. (4) Ether does not produce a sufficiently deep anæsthetic effect. Denied; objection not consistent with experience, if a sufficient doze be given. (5) Stronger preliminary excitement with ether. Excitement occurs in about the same number of cases with both anæsthetics. Dr. Julliard finds that a preliminary subcutaneous injection of morphine in most cases prevents excitement. (6) Vomiting is more common after ether. Statistics show that this occurs in 1 in 9 cases after ether, and that the proportion is about the same after chloroform. (7) Ether is inflammable. True; and therefore it should not be used where the thermo-cautery is to be employed in operations on the head. As a matter of prudence, chloroform is to be then preferred. (8) Ether causes coughing during the operation. True ; but this may be diminished by breathing at first through the nose a vapor not too concentrated, and by a subcutaneous injection of morphine. (9) Ether salivates. An exceptional phenomenon, occurring in 1 in 20 cases, and disappearing during deep anæsthesia. Where it is necessary to guard against it, a subcutaneous injection of morphine and atropine is efficacious. (10) Ether excites bronchial secretions. This occurs rarely, and the tendency is much diminished by the subcutaneous injection of morphine and by breathing diluted vapor. It occurs chiefly at the beginning of the administration, and disappears in deep anæsthesia. (11) Ether causes cyanosis. This occurs in nervous and alcoholic subjects. When observed, stop the administration, and recommence when the cyanosis has passed off. Cyanosis only occurs in cases in which any anæsthetic is more than usually dangerous. (12) Ether asphyxiates. This accident may occur in one of two ways : (*a*) by an arrest of the respiratory mechanism ; or (*b*) by tracheo-bronchial hypersecretion. It is not common. Stoppage of administration and artificial respiration are the proper procedures. (13) Vapour of ether disagreeable to other patients, nurses, etc. This is largely avoided by using a proper mask. (14) Ether causes severe muscular tremors. This occurs rarely, chiefly in alcoholics, usually in the lower limbs, ceases during deep anæsthesia, and it may pass off on strong flexion of the great toe. It is very rarely seen after subcutaneous injection of morphine. (15) Ether is eliminated more slowly. It is true that patients recover more quickly from ether than from chloroform, but occasionally cases occur in which the recovery is not complete for several hours. These cases are very rare, and have no disagreeable conclusion. The rule is rapid recovery. (16) Ether lowers the temperature more than chloroform. This is also true, but the difference is so small as to be of no account. (17) Ether causes nephritis, pulmonary congestion, bronchitis, even pneumonia. Not admitted.

Dr. Julliard, in all operations of long duration, gives a subcutaneous injection of one-sixth to one-third of a grain of morphine. He finds that this calms the patient, notably diminishes the quantity of ether necessary, and sometimes produces, after a few whiffs of ether, a remarkable condition of analgesia, or insensibility to pain without loss of consciousness. The latter condition is undoubtedly the ideal anæsthetic state. The injection is given to the patient in a quiet room, and he is encouraged to close his eyes and to sleep. In about 20 minutes he is carried to the operating table, where in quietness and without excitement he is etherized. Julliard narrates 7 cases of analgesia with ether and 1 case with chloroform. In all these cases the patient was conscious but felt no pain, and in several he was able to aid the surgeon by voluntarily making convenient movements.

In conclusion, Dr. Julliard denounces the method of giving a concentrated vapour of ether at the beginning of the inhalation, with the view of quickly producing anæsthesia, as being likely to cause any or several of the accidents of ether administration. He also views with disfavor Kocher's method of giving chloroform at the beginning, and of continuing the anæsthesia with ether, designating the method as the combination of the dangers of chloroform with the disadvantages of ether.—*The British Medical Journal for April.*

---

THE NECESSITY FOR IMMEDIATE SURGICAL INTERVENTION IN LACERATIONS OF THE PERINEUM.—The misfortune which has just happened to an unfortunate practitioner, who was mulcted damages to the amount of nine hundred dollars and costs by the Superior Court for neglecting a lacerated perineum, should be a lesson to every accoucheur in this country. It was shown that the laceration was complete, extending into the rectum, and had been followed by procidentia of the uterus and other distressing accidents.

The duty of attending to all extensive lacerations of the perineum at the time when the lesions are fresh is insisted upon by the best obstetrical writers, and it is certainly the practice of the most successful obstetricians in this country to immediately put in three or more deep stitches, thus approximating and keeping in apposition the lacerated parts till union takes place. It matters little what material is used for the sutures ; some use chromacized catgut, and make a deep continuous suture, and certainly catgut has in many instances proved to be sufficiently enduring ; others prefer silk, others silver wire. Every physician has, or ought to have in his pocket-surgical-case ligatures and curved needles, and if a sufficiency of interrupted sutures are inserted immediately after confinement, the old-fashioned quilt suture may well be dispensed with. It will not always be necessary to give the patient ether in order to insert the stitches, though some nervous and susceptible subjects may require it. It is true that after a long and difficult confinement case, the medical attendant is generally tired out and shrinks from another operation, especially where anæsthesia is required, but he must muster strength and nerve for the occasion if he would escape liability to a suit for malpractice ; and if he inserts a few stitches he will save himself from the imputation of ignorance or carelessness. Modern juries have not the name of being very tender to the feelings, reputation or pockets of physicians, and it goes without saying that the most vigilant and attentive will be the least likely to be "caught napping."

There are one or two errors that should be cleared away, lest they should be subterfuges for the careless. The one is that tying the knees of the patient together will answer the same end as sutures. "Only a very credulous person," says Lusk, "really believes that he has witnessed union by first intention in extensive ruptures as the result of tying the knees together, and enjoining rest upon the side. The action of the transversi-perinei muscles tends to draw the torn surface apart. Moreover, the necessity of separating the knees in passing urine, and to enable the nurse to clean the genitalia, makes it impossible to keep them in contact for any lengthened period."

The other mistaken notion is that primary perineorraphy rarely succeeds, "that the perineum is not merely torn but is contused and mangled and that the previously œdematous and infiltrated tissues are predisposed to gangrene, and consequently are in the worse possible condition for immediate union." (Charpentier.) Moreover, it is said that the lochial discharge will always be an obstacle to union by first intention.

According to the experience of very many who have tried the immediate operation, and who have seldom or never failed to obtain good union, if due pains toward cleanliness and antisepsis are taken, no such unfavorable result as Charpentier points out need ever be feared. Certainly, Charpentier's American editor warmly favors the immediate repair of any laceration beyond the first degree, for the reason that thus a possible entrance site for septic matter is prevented, and also because the operation is a simple affair after delivery, and more extensive and complicated, the longer we wait. He recommends that in case of laceration to the second degree only one deep silk or wire suture should be used; if the rent be deeper, three to five will be needed. In any event the patient should be placed on her side, a wad of absorbent cotton inserted into the vagina to catch the discharges, the wound carefully washed and trimmed of jagged shreds, and then, guided by the finger in the rectum, the suture is passed deeply around, at one-half inch from the margins. The line of suture should be dusted with iodoform, and a narrow strip of cotton laid along the perineum and the posterior vaginal wall. The after-treatment will consist in dusting with iodoform twice daily, and replacing the strip of cotton by a fresh piece till the six or seventh day, when the sutures may be removed.—*Boston Medical and Surgical Journal for May.*

---

TREATMENT OF MALIGNANT NEOPLASMS NOT AMENABLE TO OPERATION.—Prof. v. Mosetig-Moorhof, of Vienna, states (*Wiener Med. Presse* 6, '91, as quoted by *Pittsburg Med. Rev.*, May, 1891) that he had employed all recommended remedies for years without noteworthy result. But he kept in mind that the pathogenic cell-elements possess decidedly inferior biological potency to the healthy tissue elements—pointing to the possibility of making active warfare upon the neoplasm without affecting the surrounding healthy tissue. Proliferation of the pathological cell-elements, upon which the growth of the neoplasm depends, occurs from the nucleus of the mass. Hence, Mosetig thought, to concentrate treatment upon the proliferating nucleus would arrest the process, and even induce retrograde metamorphosis. This led him to stain the neoplastic tissue—an easy task—by filling it with aniline dye freed from arsenic. His first experimental case was a man, age 50, with an orange-sized, *round-cell sarcoma, in the inguinal region*, which several prominent Vienna surgeons pronounced unfit for operation. Mosetig injected one gram of a one per cent. solution of *aniline trichlorate* into the sarcomatous mass. After eight weeks treatment, the tumor diminished to size of a hickory-nut, with a healthy cicatrix at the site of the ulcer, and the patient was discharged decidedly improved. A year later, the man died of pneumonia, *without even a sign of recurrence* of the growth. Mosetig employed aniline trichlorate in three other cases, but was obliged to discontinue its use because of unpleasant effect in other directions.

A year ago, two new dyes—*methyl-violet* and pyoktanin—were introduced, and said to be perfectly harmless by Prof. Stilling. Mosetig selected a lady, age 60, with a *sarcoma of inferior maxilla*, size of a fist, filling the oral cavity, and forcing the tongue up against the hard palate, so that she could neither speak nor swallow. The growth was injected with methyl-violet solution 1:500, which was increased to 1:300. In all, 35 injections were given of from 3 to 6 grams of solution at each sitting. Then the growth had shruken so that only a portion of the interosseous enlargement remained, and the patient was free of suffering. Up to this writing, no malignant disposition is manifest.

Five other cases—*cysto-sarcoma of sterno-clavicular joint, papilloma of urinary bladder, sarcoma of peritoneum* and two *carcinomata of cervical glands*—have all done equally well with methyl-violet injections—all being decidedly improved, with possible absolute cure in the near future. Tumors not suppurating do not degenerate, but simply shrink together in retrogressive metamorphosis; while such as are ulcerated and discharing pus for a time suppurate more freely, after which, with some diminution in size, they cicatrize rapidly.

The injections are repeated every two or three days, and made under strict aseptic precautions. Thus far, Mosetig has employed solutions of the strength of 1:1000, 1:500, 1:300; and he is of opinion that much stronger solutions may be used without danger.—*Va. Med. Monthly.*

---

THE COLITIS OF INFANTS.—Dr. James M. French, in his valuable contribution (*Journal of the American Medical Association*), gives the following dietetic and medicinal treatment for colitis of infants: The child must receive the proper quantity of the right kind of food at the right intervals for its age. Not seldom the error will be found to consist in the too early resort to a mixed diet, too frequent nursing, or the use of such inferior substitutes for mother's milk as impure cow's milk, condensed milk, or an inferior quality of artificial food, or the use of improperly prepared food. The diet should consist of articles of food which are most certain to undergo easy and complete digestion, leaving as little residue as possible. The passage of healthy fæces from the small intestine into the larger in these cases is sufficient to excite peristalsis. For this reason overfeeding must be guarded against.

Ordinarily, the diet of nursing infants may be restricted to the mother's milk, and that of infants that have been weaned, to sterilized cow's milk. In severe cases, however, it is necessary to discontinue even cow's milk for a time. By this means the inflamed bowel is freed from the influences which keep up the inflammation. Something must be given both to provide nourishment and to satisfy thirst; for this the author highly indorses Mellin's Food, prepared with *water instead of milk*, as it furnishes ample nutriment and leaves almost no residue in the bowel. In addition to this, an occasional teaspoonful of freshly expressed beef juice and a few drops of brandy may be given.

The writer rarely employs any medicines other than those contained in the following prescriptions :

R. Pepsinæ (F. & F.), gr. xii to xxiv.
Hydrarg. chlor. mit., gr. ss to j.
Sacch. lactis., q. s.
M. et ft. chart. No., xii.

SIG.—One powder every three hours after nursing.

R. Ex. Pancreatis (F. & F.), ʒss to j.
Hydrarg. chlor. mit., gr. ss to j.
Sacch. lactis, q. s.
M. et ft. chart., No. xii.

SIG.—One powder every three hours immediately before or after nursing.—*Annals of Gynecology and Pediatry.*

---

ARISTOL.—Aristol (the bichloride of dithymol) is rapidly coming into favor as an excellent substitute for iodoform. It possesses all the therapeutic properties of iodoform without having the inconveniences of its irritant and poisonous action. It is a reddish-brown amorphous powder, becoming more and more pale as it looses its iodine from exposure to heat or light. It is soluble in water or glycerine, slightly so in alcohol, and very soluble in ether, chloroform and liquid vaseline. The powdered aristol has already rendered great service as a cicatrizing agent in ulceration of the skin and mucous membranes, in epitheliomata, in ulcers of the leg, and in tuberculosis and syphilitic ulcerations. Its application to these ulcerations is not painful, nor does it produce any of the phenomena of general poisoning, as does iodoform. Under the form of a pomade containing from ten to twenty parts of aristol to one hundred of vaseline, it has been used in cutaneous diseases, such as psoriasis, eczema, lupus, etc. Some observers have employed it in diseases of the nose, throat and larynx, and it has been used in gynæcological practice in the treatment of endometritis, erosions of the cervix, and vulvar eczema. Swieciki has made pencils and suppositories thus:

℞. Aristol. ................................ ........grs., 80.
Gum Arabic, a sufficient quantity to make
five pencils. And

℞. Aristol ........ ...........................grs., 7½ to 15.

Ol. theobrom., sufficient to make one vaginal suppository.

It may be used in ethereal solution, or in collodion if so desired.

Internally it has been given in pill form associated with the hypophosphite of sodium in cases of fœtid bronchitis and gangrene of the lung, with excellent results. In four or five days the sputa loose their offensive odor and the general condition is greatly improved. In pulmonary phthisis it diminishes the amount of expectoration.—*Huchard, in Revue Gen. de Clin. et de Therapeutique, January* 14, 1891.

---

CHLORALAMID AS A HYPNOTIC.—Dr. G. Generisch has prescribed chloralamid in 32 cases, giving 30 grains at night. This dose was generally sufficient to induce sleep within half an hour. A more certain effect and a longer sleep was obtained when 45 or 60 grains were prescribed. He considers choloralamid preferable to other hypnotics, both because it acts more decidedly and because it is less unpleasant to take. It must be remembered that its effect is negative when sleeplessness is due to pain. It is not by any means a dangerous drug, but headache and vomiting may occur after a very large dose. It does not seem to affect the digestion nor the renal functions. The pulse generally becomes softer and more frequent.—*Med. Rec.*

---

TREATMENT OF INFANTILE ECZEMA CAPITIS.—Correct the acidity of the stomach by alkalies, and a properly regulated diet. A powder consisting of two grains each of calomel and bicarbonate of soda may be given about every other day; or a little Rochelle salts may be added. After the crusts have been removed by the local application of vaseline the following ointment should be constantly applied:

℞. Camphor, gr. x.

Calomel, } aa. ʒi.

Goulard's Extract, }

Vaseline, ℥i.

—*R. W. Taylor, M. D., Medical News.*

AVOIDANCE OF STIMULANTS DURING HEMORRHAGE.—It is customary, when the accident of hemorrhage occurs, for the operator, or some bystander, to administer wine, brandy, or some other alcoholic stimulant to the patient, under the false idea of sustaining the vital power. It is my solemn duty to protest against this practice on the strictest and purest scientific grounds. The action of alcohol, under such circumstances, is injurious all round. It excites the patient, and renders him or her nervous and restless. It relaxes the arteries, and favors the escape of blood through the divided structures. Entering the circulation in a diluted state, it acts after the manner of a salt in destroying the coagulating quality of the blood ; and above all other mischiefs, it increases the action of the heart, stimulating it to throw out more blood through the divided vessels. These are all serious mischiefs, but the last named is the worst. In hemorrhage the very keystone of success lies so much in quietness of the circulation that actual failure of the heart, up to faintness, is an advantage, for it brings the blood at the bleeding point to a standstill, enables it to clot firmly, when it has that tendency, and forms the most effective possible check upon the flow from the vessels. (The author refers to a case in which three pounds of blood was lost and the patient was unconscious, but which recovered. He refers to this case as typical, because, if a stimulant were not wanted in it, a stimulant cannot be called for in examples less severe.) The course followed was simply to lay the patient quite recumbent when signs of faintness supervened, and, so long as he could swallow, to feed him with warm milk and water freely. Such, in my opinion, is the proper treatment to be employed in every instance of syncope from loss of blood.
—*The Asclepiad, Dietetic Gazette.*

---

FIFTEENTH ANNUAL MEETING OF THE AMERICAN DERMATOLOGICAL ASSOCIATION, TO BE HELD AT THE SHOREHAM HOTEL, WASHINGTON, D. C., SEPTEMBER 22d, 23d 24th AND 25th, 1891.—*Officers for* 1891: President—F. B. Greenough, M. D., of Boston; Vice-President—L. N. Denslow, M. D., of

St. Paul; Secretary and Treasurer—George Thomas Jackson, M. D., of New York. *Programme of Papers:* 1. Dermatitis Hæmostatica—Dr. H. G. Klotz. 2. A case of Lupus Erythematosus with fatal complications—Dr. W. A. Hardaway. 3. Report of a case of Universal Erythema Multiforme, with colored portrait and specimen—Dr. L. A. Duhring. 4. An unusual case of Sarcoma involving the skin of the arm, amputation, recovery—Dr. F. J. Shepherd. 5. Multiple Sarcomata. History of a case showing modification, and amelioration of symptoms with large doses of arsenic—Dr. S. Sherwell. *Discussion on Tuberculosis of the Skin:* 6. Its Clinical aspects and relations—Dr. J. C. White. 7. Its Pathology—Dr. J. T. Brown. 8. Its Treatment—Dr. G. H. Fox. 9. Thirteen cases of Tuberculosis of the skin, with their treatment—Dr. J. S. Howe. 10. A case of Lichen Scrofulosorum—Dr. J. Grindon. 11. Notes of a visit to the Leper Hospital at San Remo, Italy, with photographs—Dr. L. A. Duhring. 12. The treatment of Alopecia Areata—Dr. P. A. Morrow. 13. A Therapeutic note on Alopecia. Areata—Dr. L. D. Bulkley. 14. Morphœa Atrophica of Wilson—Dr. R. W. Taylor. 15. The treatment of Pruritus—Dr. E. B. Bronson. 16. Prairie Itch—Dr. L. N. Denslow. 17. Diseases of the skin associated with derangements of the nervous system—Dr. W. T. Corlett. 18. Treatment of Chronic Ringworm in an Institution for boys—Dr. L. A. Duhring. 19. Notes of a case of acute Dermatitis Exfoliativa—Dr. J. E. Graham. 20. Note relative to Pemphigus Vegetans—Dr. J. N. Hyde. 21. A study of Mycosis Fungoides with report of a case—Drs. H. W. Stelwagon and H. Leffingwell Hatch. 22. Lymphangioma Circumscriptum with report of a case—Dr. M. B. Hartzell. 23. Remarks on Carbuncle with report of a peculiar case—Dr. H. G. Klotz. 24. Note on Erythema et Nævus Nuchæ—Dr. C. W. Allen. 25. A case of Lichen Ruber—Dr. J. Grindon. 26. The personal equation in Dermatology—Dr. L. D. Bulkley. 27. The hypodermic use of Hydrargyrum Formamidatum in syphilis—Dr. R. B. Morrison. 28. Retarded hereditary syphilis—Dr. R. B. Morrison. 29. Epilation, its range of usefulness as a dermato-therapeutic measure—Dr. J. Zeisler.

